# E-cigarette use among individuals with chronic obstructive pulmonary disease

**DOI:** 10.1007/s11739-026-04385-z

**Published:** 2026-05-16

**Authors:** Sultan Alhabdan, Abdalkareem Alashjaai, Yusuff Adebayo Adebisi

**Affiliations:** 1 Ad Diriyah Hospital, Third Health Cluster, Ministry of Health, Riyadh, Saudi Arabia; 2 College of Social Sciences, University of Glasgow, Glasgow, UK

**Keywords:** Chronic obstructive pulmonary disease, E-cigarettes, Smoking cessation, Harm reduction, Socioeconomic deprivation, Tobacco use, Survey methods, Scotland

## Abstract

Chronic obstructive pulmonary disease (COPD) is strongly linked to tobacco smoking, and smoking cessation is central to disease management. E-cigarettes are widely promoted as a harm reduction option in the United Kingdom, yet patterns of use among people with COPD remain poorly described. This study examined the prevalence and correlates of current e-cigarette use among individuals with COPD in Scotland using data from the 2018 to 2022 Scottish Health Survey. The analysis included adults aged 16 years and above who reported a COPD diagnosis (*n* = 891). Survey-weighted logistic regression was used to estimate crude and adjusted associations between sociodemographic, behavioural, and health characteristics and current e-cigarette use, with additional models stratified by smoking status and smoking intensity. The weighted prevalence of current e-cigarette use was 11.9% (95%CI 9.7–14.5). Older age was associated with lower odds of current use (adjusted odds ratio [aOR] 0.60; 95%CI 0.41–0.89), while socioeconomic deprivation showed a positive gradient (aOR 1.21; 95%CI 1.01–1.45). Smoking status was the strongest correlate: odds were higher among ex-smokers (aOR 10.80; 95%CI 1.66–70.06) and current smokers (aOR 14.86; 95%CI 2.37–93.33) compared with never-smokers. Among current smokers, younger age, female sex, and higher deprivation were associated with use, whereas no clear determinants were identified among former smokers. Light and moderate smokers had greater odds of current e-cigarette use than non-smokers, while heavy smokers did not differ. Overall, e-cigarette use was relatively common and concentrated among younger, more deprived adults with a smoking history. Longitudinal studies are needed to determine the implications for respiratory outcomes.

## Introduction

Chronic obstructive pulmonary disease (COPD) is a major cause of morbidity and mortality worldwide and represents a substantial public health burden in the United Kingdom [1]. In the UK, over 1.2 million people were estimated to have a diagnosed case of COPD in 2022, with the country having one of the highest mortality rates from the disease in Europe [1]. Characterised by persistent respiratory symptoms and airflow limitation, COPD is strongly linked to long-term tobacco smoking, which remains the primary modifiable risk factor [2]. Although smoking prevalence has declined in recent decades, people living with COPD continue to smoke at disproportionately high rates [3], contributing to poorer symptom control, accelerated lung function decline, and higher rates of hospital admission. As a result, promoting smoking cessation remains central to COPD management and national respiratory health strategies [4].

In recent years, e-cigarettes have emerged as a widely used alternative to combustible tobacco within the UK [5–7]. Evidence suggests that e-cigarettes deliver nicotine with markedly fewer toxicants than cigarette smoke and may support smokers in reducing or quitting cigarette use [8–10]. The UK clinical and regulatory context is distinctive in encouraging the use of e-cigarettes for harm reduction, with national guidance highlighting their potential role in helping smokers who struggle to quit using traditional methods [11–13]. Despite this supportive policy environment, the profile of e-cigarette use among people with COPD has been insufficiently characterised at the population level.

Existing research in COPD populations has focused mainly on clinical outcomes, cessation support, and attitudes toward smoking cessation [14–18], with far less attention given to identifying who within this group engages in current e-cigarette use. Understanding sociodemographic, behavioural, and health-related factors associated with e-cigarette use is critical for informing targeted harm reduction strategies and for ensuring that cessation services align with the needs of individuals living with chronic respiratory disease. Moreover, given the wide socioeconomic inequalities in COPD prevalence and severity, identifying patterns of e-cigarette use across demographic groups may help guide equitable policy and clinical practice.

This study aimed to investigate the prevalence and correlates of current e-cigarette use among individuals with COPD using nationally representative data from the Scottish Health Survey. Specifically, we examined sociodemographic characteristics, smoking behaviours, and health indicators associated with current e-cigarette use, including stratified analyses by smoking status and an assessment of smoking intensity.

## Methods

### Data source and study design

This study used data from the 2018, 2019, 2021 and 2022 waves of the Scottish Health Survey (SHeS), a nationally representative, cross-sectional survey of individuals living in private households across Scotland. Across the four survey years combined, the dataset included 25,986 participants, with annual sample sizes of 6790 in 2018, 6881 in 2019, 6157 in 2021, and 6158 in 2022. The 2020 SHeS wave, collected during the COVID-19 pandemic and designated as experimental statistics, was excluded, because its methodology differed substantially from other years and was not considered comparable [6]. The SHeS is designed to monitor the health, wellbeing, and health-related behaviours of the Scottish population and to support national public health surveillance. It employs a multistage, stratified probability sampling strategy based on the Postcode Address File, ensuring both geographical and demographic representativeness [6, 19]. Owing to the repeated cross-sectional design, different private households and individuals are sampled in each survey year. Data collection was undertaken through interviewer-administered, computer-assisted personal interviews, with more sensitive items completed by participants using a self-completion module. For the present study, data from the four eligible survey years were pooled to maximise sample size, increase statistical precision, and provide more stable estimates of associations related to e-cigarette use among individuals with chronic obstructive pulmonary disease.

### Study population

The study population consisted of adults aged 16 years and above from the 2018, 2019, 2021 and 2022 waves of the SHeS. After excluding respondents who were not eligible for the adult questionnaire, a total of 18,664 adults remained. COPD status was defined using the variable COPD, which identifies whether participants reported ever having been diagnosed with chronic obstructive pulmonary disease. Individuals who answered “Yes” were included, while those who responded “No”, “Don’t know”, or “Refused” were excluded. This resulted in a final analytic sample of 891 individuals with COPD.

### Outcome variable: current e-cigarette use

The primary outcome was current e-cigarette use, derived from the SHeS variable Ecigtot16, which categorises respondents as current users, former users, or never users of e-cigarettes or vaping devices. This variable is based on the survey question: “Do you use an e-cigarette or vaping device at all nowadays?” The accompanying interviewer guidance note specifies that an e-cigarette or vaping device “is a device that uses a battery to create a vapour” and that, unlike normal cigarettes, such devices “do not burn, nor contain tobacco”, thereby distinguishing e-cigarettes from other nicotine products. For this analysis, a binary variable was created in which participants were classified as current e-cigarette users if they reported using an e-cigarette at the time of the survey (Ecigtot16 = 1), and as non-users if they reported former use or never use (Ecigtot16 = 2 or 3). The final coded variable (ecig_now), therefore, distinguished current use (1 = Yes) from non-use (0 = No).

### Predictor variables

Predictor variables included sociodemographic, behavioural, and health-related factors informed by prior evidence on smoking and nicotine use in chronic respiratory disease [20–22]. Age was grouped into three categories (16–44, 45–64, and 65 years and above), and sex was classified as male or female. Socioeconomic position was measured using national Scottish Index of Multiple Deprivation (SIMD) quintiles, ranging from least deprived to most deprived. Educational attainment was categorised as higher education, further or upper secondary education, and lower or no qualifications. Smoking status distinguished never-smokers, former smokers, and current smokers, and, in a secondary analysis, smoking intensity was examined using categories of light, moderate, heavy, and unknown number of cigarettes smoked per day with non-smokers as the reference group. Self-rated general health was grouped into very good or good, fair, and bad or very bad, while alcohol consumption was classified as never, ex-drinker, or current drinker. Survey year was included as a categorical covariate to account for temporal differences across the 2018, 2019, 2021, and 2022 waves.

### Statistical analysis

All analyses accounted for the complex multistage sampling design of the Scottish Health Survey by incorporating survey weights, primary sampling units, and strata using Stata’s svy commands. The survey design comprised 14 strata and 625 primary sampling units. Design effects for the primary predictors ranged from 1.05 to 1.47, indicating minimal impact of clustering on variance estimation.

Descriptive statistics were used to summarise the characteristics of individuals with COPD. Weighted prevalence estimates of current e-cigarette use were calculated using the survey weight, while the distributions of sociodemographic, behavioural, and health-related characteristics were presented using unweighted counts and percentages. Differences between current e-cigarette users and non-users were assessed using design-adjusted F-statistics with Rao–Scott correction to account for the complex survey design.

Survey-weighted logistic regression models were used to examine crude and adjusted associations between the independent variables and current e-cigarette use. The adjusted model included age, sex, socioeconomic deprivation, educational level, smoking status, self-rated general health, alcohol consumption, and survey year (2018–2022). Age and deprivation were modelled as ordered continuous variables. Orthogonal polynomial contrast tests supported the linear specification for deprivation (linear *p* = 0.004). For age, polynomial contrasts indicated a non-linear association (quadratic *p* = 0.049); categorical results are, therefore, also presented alongside the continuous specification to fully characterise the age pattern. To assess temporal heterogeneity, design-adjusted Wald tests were used to examine interactions between survey year and each of the three principal predictors (age, socioeconomic deprivation, and smoking status), with non-significant interaction terms supporting the inclusion of survey year as a categorical covariate rather than as an effect modifier. Results are presented as odds ratios (ORs) with 95% confidence intervals (CIs) and *p* values.

To explore whether associations differed by smoking history, separate survey-weighted logistic regression models were fitted for current smokers and former smokers. In a secondary analysis, the association between smoking intensity and current e-cigarette use was examined in a separate model, with current smokers categorised as light (< 10/day), moderate (10–19/day), heavy (≥ 20/day), or unknown cigarettes per day, and non-smokers as the reference group.

Sensitivity analyses were conducted in three ways. First, the sample was restricted to ever-smokers (excluding never-smokers), given the very low prevalence of current e-cigarette use among those who had never smoked. The same fully adjusted model was fitted using the svy, subpop() approach to ensure correct variance estimation. Second, Firth’s penalised logistic regression was used to assess whether the primary fully adjusted estimates were affected by sparse-data bias, given the small number of never-smokers reporting current e-cigarette use (*n* = 2). Firth’s method applies a Jeffreys prior to the likelihood, reducing small-sample bias and ensuring finite parameter estimates in the presence of rare events or quasi-complete separation. As Firth’s method does not accommodate complex survey designs, this analysis serves as a robustness check for point estimates rather than a replacement for the primary survey-weighted models. Third, given that the outcome prevalence (11.9%) was sufficiently high that odds ratios may overstate the magnitude of associations relative to prevalence ratios, we re-estimated the fully adjusted primary model using a modified Poisson regression with survey-design robust (Taylor-linearised) variance to obtain prevalence ratios (PRs) with 95% confidence intervals.

All analyses were conducted in Stata 18. A two-sided *p* value of less than 0.05 was considered statistically significant.

## Results

### Prevalence of current e-cigarette use

Among the 891 individuals with COPD included in the analysis, the weighted prevalence of current e-cigarette use was 11.9% (95%CI 9.7–14.5%), while 88.1% (95%CI 85.5–90.3%) were non-users.

### Characteristics of individuals with COPD by e-cigarette use

Table 1 presents the distribution of sociodemographic, behavioural, and health characteristics by current e-cigarette use status. Age differed significantly between users and non-users. More than half of current e-cigarette users were aged 45–64 years (51.4%), compared with 31.3% of non-users, while a smaller proportion of users were aged 65 years and above (44.0%) compared with non-users (63.9%). Women accounted for a greater proportion of current users (66.1%) compared with non-users (55.0%), although this difference was not statistically significant after accounting for the survey design. Socioeconomic deprivation showed a significant gradient, with current use more common in the most deprived quintiles. For example, 33.9% of current users were in the second most deprived quintile compared with 22.8% of non-users. Educational attainment did not differ significantly between groups, with lower or no qualifications being the most common category among both users (70.6%) and non-users (65.7%). Smoking status showed the strongest association with current e-cigarette use. Over half of current users were current cigarette smokers (56.9%), compared with one-third of non-users (33.4%). Very few never-smokers reported e-cigarette use (1.8%). Alcohol consumption was not associated with current e-cigarette use. Approximately two-thirds of both users (70.6%) and non-users (66.9%) reported current drinking. Self-rated health did not differ significantly between groups, although a higher proportion of current users reported bad or very bad health (51.4%) compared with non-users (40.0%).

**Table 1:** Weighted prevalence and unweighted characteristics of individuals with COPD by current e-cigarette use (*n* = 891)

Variable	Non-user (*n* = 782)	Current user (*n* = 109)	Total (*n* = 891)	*F*, *p* value
Weighted prevalence	88.1% (95%CI 85.5 to 90.3)	11.9% (95%CI 9.7 to 14.5)		
Age group				*F*(2.00, 1220.36) = 8.64, *p* < 0.001
16–44	37 (4.7%)	5 (4.6%)	42 (4.7%)	
45–64	245 (31.3%)	56 (51.4%)	301 (33.8%)	
65 +	500 (63.9%)	48 (44.0%)	548 (61.5%)	
Sex				*F*(1, 611) = 1.54, *p* = 0.215
Male	352 (45.0%)	37 (33.9%)	389 (43.7%)	
Female	430 (55.0%)	72 (66.1%)	502 (56.3%)	
SIMD 2020 quintile				*F*(3.70, 2259.44) = 3.96, *p* = 0.004
Least deprived	92 (11.8%)	5 (4.6%)	97 (10.9%)	
4th	129 (16.5%)	9 (8.3%)	138 (15.5%)	
3rd	135 (17.3%)	22 (20.2%)	157 (17.6%)	
2nd	178 (22.8%)	37 (33.9%)	215 (24.1%)	
Most deprived	248 (31.7%)	36 (33.0%)	284 (31.9%)	
Education				*F*(2.00, 1220.45) = 1.41, *p* = 0.244
Higher	146 (18.7%)	19 (17.4%)	165 (18.5%)	
Further/upper secondary	122 (15.6%)	13 (11.9%)	135 (15.2%)	
Lower/none	514 (65.7%)	77 (70.6%)	591 (66.3%)	
Smoking status				*F*(2.00, 1219.31) = 10.61, *p* < 0.001
Current smoker	261 (33.4%)	62 (56.9%)	323 (36.2%)	
Former smoker	374 (47.8%)	45 (41.3%)	419 (47.0%)	
Never smoked	147 (18.8%)	2 (1.8%)	149 (16.7%)	
Alcohol consumption				*F*(2.00, 1221.41) = 0.48, *p* = 0.620
Never	67 (8.6%)	9 (8.3%)	76 (8.5%)	
Ex-drinker	192 (24.6%)	23 (21.1%)	215 (24.1%)	
Current drinker	523 (66.9%)	77 (70.6%)	600 (67.3%)	
Self-rated health				*F*(1.93, 1178.47) = 2.38, *p* = 0.094
Very good/good	187 (23.9%)	20 (18.4%)	207 (23.2%)	
Fair	282 (36.1%)	33 (30.3%)	315 (35.4%)	
Bad/very bad	313 (40.0%)	56 (51.4%)	369 (41.4%)	

Weighted prevalence estimates of current e-cigarette use were derived using the Scottish Health Survey complex survey design, applying primary sampling units, strata, and sampling weights

Counts and percentages within each characteristic are unweighted and represent the proportion of individuals in that category among current users and non-users, respectively

Design-adjusted F-statistics with Rao–Scott correction were used to test group differences, accounting for the complex survey design

Totals represent complete-case counts for each variable

Table 2 shows the crude and adjusted associations between participant characteristics and current e-cigarette use among individuals with COPD. Older age was associated with substantially lower odds of current e-cigarette use, with each step into an older age category linked to a 40% reduction in odds (aOR 0.60; 95%CI 0.41–0.89; *p* = 0.011). When modelled categorically, neither age group showed a statistically significant association compared with those aged 16–44 (45–64: OR 1.64, 95%CI 0.54–5.00; 65 + : OR 0.71, 95%CI 0.23–2.18), likely reflecting limited statistical power within age strata. Socioeconomic deprivation demonstrated a positive gradient, whereby individuals in more deprived areas had higher odds of current use (aOR 1.21; 95%CI 1.01–1.45; *p* = 0.042). Women had slightly higher odds of e-cigarette use compared with men, although this association was imprecise (aOR 1.44; 95%CI 0.87–2.40; *p* = 0.160). Educational level was not clearly associated with current use, with confidence intervals for both higher education (aOR 1.15; 95%CI 0.57–2.30; *p* = 0.696) and further/upper secondary education (aOR 0.57; 95%CI 0.27–1.22; *p* = 0.147) spanning unity. Smoking status remained the strongest correlate: compared with never-smokers, ex-smokers had markedly higher odds of current e-cigarette use (aOR 10.80; 95%CI 1.66–70.06; *p* = 0.013), and the odds were even greater among current smokers (aOR 14.86; 95%CI 2.37–93.33; *p* = 0.004). Neither self-rated health nor alcohol consumption showed clear associations with e-cigarette use, with wide confidence intervals that included the null value.

**Table 2:** Crude and adjusted associations with current e-cigarette use (*n* = 891)

Variable	Crude OR (95%CI), *p* value	Adjusted OR (95%CI), *p* value
Age group (continuous: 16–44 → 45–64 → 65 +)	0.65 (0.47–0.90), *p* = 0.009	0.60 (0.41–0.89), *p* = 0.011
Sex		
Male	Reference	Reference
Female	1.36 (0.83–2.23), *p* = 0.216	1.44 (0.87–2.40), *p* = 0.160
Deprivation (continuous, least → most deprived quintile)	1.30 (1.11–1.52), *p* = 0.001	1.21 (1.01–1.45), *p* = 0.042
Educational level		
Higher	0.79 (0.34–1.88), *p* = 0.600	1.15 (0.57–2.30), *p* = 0.696
Further/upper secondary	1.35 (0.74–2.47), *p* = 0.326	0.57 (0.27–1.22), *p* = 0.147
Lower/none	Reference	Reference
Smoking status		
Never smoker	Reference	Reference
Ex-smoker	9.29 (1.61–53.53), *p* = 0.013	10.80 (1.66–70.06), *p* = 0.013
Current smoker	17.25 (3.03–98.16), *p* = 0.001	14.86 (2.37–93.33), *p* = 0.004
Self-rated health		
Very good/good	Reference	Reference
Fair	1.22 (0.64–2.33), *p* = 0.552	1.13 (0.59–2.18), *p* = 0.714
Bad/very bad	1.85 (1.04–3.30), *p* = 0.037	1.49 (0.78–2.88), *p* = 0.230
Alcohol consumption		
Never	Reference	Reference
Ex-drinker	0.98 (0.39–2.47), *p* = 0.961	0.79 (0.30–2.10), *p* = 0.640
Current drinker	1.26 (0.54–2.90), *p* = 0.593	1.31 (0.54–3.21), *p* = 0.549

Age and deprivation (SIMD) were modelled as continuous variables, with higher values indicating older age groups (16–44 → 45–64 → 65 +) and greater socioeconomic deprivation (1 = least deprived to 5 = most deprived)

Educational level, smoking status, self-rated health, alcohol consumption, and sex were treated as categorical variables, with reference categories shown in the table

All adjusted models account for age group, sex, SIMD deprivation, educational level, smoking status, self-rated health, alcohol consumption, and survey year (2018–2022)

OR > 1 indicates higher odds of current e-cigarette use; OR < 1 indicates lower odds

Table 3 shows the adjusted associations between participant characteristics and current e-cigarette use stratified by smoking status. Among current smokers, older age was associated with lower odds of current e-cigarette use (aOR 0.54; 95%CI 0.33–0.90; *p* = 0.019). Women had higher odds of use compared with men (aOR 2.53; 95%CI 1.28–5.00; *p* = 0.008). Socioeconomic deprivation was also positively associated with current use, with individuals living in more deprived areas having increased odds (aOR 1.39; 95%CI 1.03–1.88; *p* = 0.034). In contrast, none of the examined characteristics were significantly associated with current e-cigarette use among former smokers, with all confidence intervals crossing unity.

**Table 3:** Adjusted associations with current e-cigarette use stratified by smoking status

Variable	Current smokers (*n* = 323) Adjusted OR (95%CI), *p* value	Former smokers (*n* = 419) Adjusted OR (95%CI), *p* value
Age group (continuous: 16–44 → 45–64 → 65 +)	0.54 (0.33–0.90), *p* = 0.019	0.61 (0.34–1.12), *p* = 0.110
Sex		
Male	Reference	Reference
Female	2.53 (1.28–5.00), *p* = 0.008	1.00 (0.52–1.91), *p* = 0.989
Deprivation (continuous, least → most deprived quintile)	1.39 (1.03–1.88), *p* = 0.034	1.16 (0.90–1.50), *p* = 0.256
Education		
Higher	2.20 (0.76–6.42), *p* = 0.146	0.75 (0.26–2.13), *p* = 0.588
Further/upper secondary	0.82 (0.31–2.17), *p* = 0.689	0.50 (0.14–1.72), *p* = 0.270
Lower/none	Reference	Reference
Self-rated health		
Very good/good	Reference	Reference
Fair	1.86 (0.62–5.59), *p* = 0.268	0.72 (0.28–1.83), *p* = 0.489
Bad/very bad	1.54 (0.55–4.29), *p* = 0.412	1.51 (0.61–3.69), *p* = 0.370
Alcohol consumption		
Never	Reference	Reference
Ex-drinker	0.86 (0.22–3.47), *p* = 0.836	0.54 (0.14–2.09), *p* = 0.367
Current drinker	2.40 (0.65–8.81), *p* = 0.186	0.65 (0.19–2.24), *p* = 0.493

Models were stratified by smoking status to assess whether determinants of current e-cigarette use differ between current smokers and former smokers

All models were adjusted for age group (continuous), sex, SIMD deprivation (continuous), educational level, self-rated health, alcohol consumption, and survey year (2018–2022)

Estimates were derived using the Scottish Health Survey complex survey design, incorporating sampling weights, strata, and primary sampling units

Odds ratios > 1 indicate higher odds of current e-cigarette use within each smoking-status stratum

**Fig. 1:**
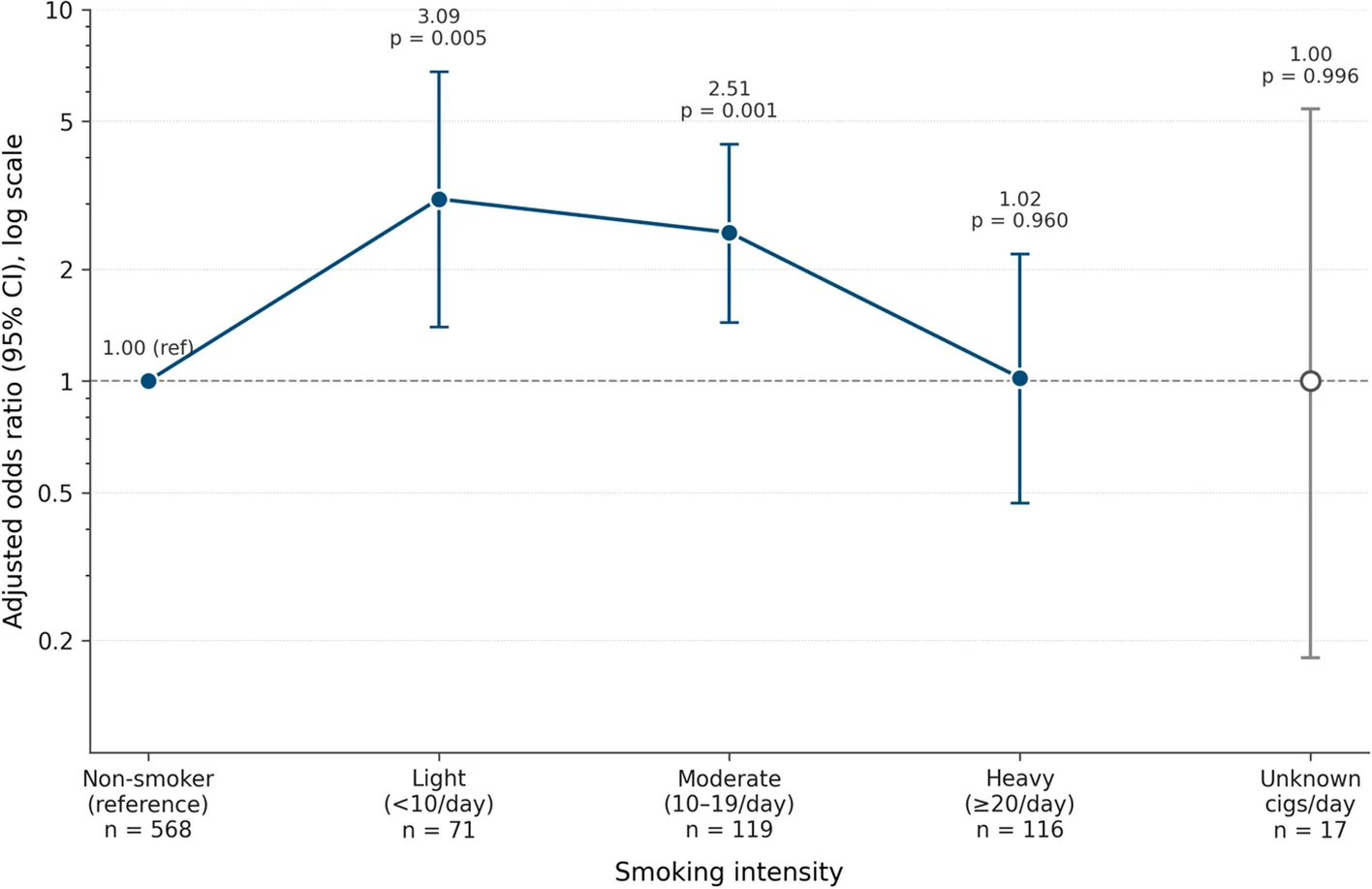
Adjusted Association between smoking intensity and current e-cigarette use (*n* = 891). Smoking intensity was analysed using four categories among current smokers, with non-smokers (former and never-smokers; *n* = 568) included as the reference group. Current smokers were classified as light smokers (< 10 cigarettes/day; *n* = 71), moderate smokers (10–19 cigarettes/day; *n* = 119), heavy smokers (≥ 20 cigarettes/day; *n* = 116), and those with unknown cigarettes per day (*n* = 17). The analytic sample comprised 891 respondents. Estimates were adjusted for age group, sex, socioeconomic deprivation (SIMD), educational level, self-rated health, alcohol consumption, and survey year (2018–2022). Odds ratios were derived using the Scottish Health Survey complex survey design. OR > 1 indicates higher odds of current e-cigarette use

Figure 1 shows the association between smoking intensity and current e-cigarette use among individuals with COPD. Light smokers (< 10/day) had more than threefold higher odds of current e-cigarette use compared with non-smokers (aOR 3.09; 95%CI 1.40–6.81; *p* = 0.005). Moderate smokers (10–19/day) also had significantly higher odds (aOR 2.51; 95%CI 1.44–4.35; *p* = 0.001). In contrast, heavy smokers (≥ 20/day) showed no clear association with current use (aOR 1.02; 95%CI 0.47–2.20; *p* = 0.960), and those who did not report the number of cigarettes smoked per day also showed no evidence of an association (aOR 1.00; 95%CI 0.18–5.39; *p* = 0.996).

### Sensitivity analysis

In a sensitivity analysis excluding never-smokers, the overall pattern of associations remained consistent with the main model. Older age continued to be associated with lower odds of current e-cigarette use (aOR = 0.53, 95%CI 0.35–0.80, *p* = 0.002). Socioeconomic deprivation remained positively associated with current use (aOR = 1.24, 95%CI 1.02–1.50, *p* = 0.027). Sex, education, self-rated health, and alcohol consumption were not statistically significant predictors in the restricted sample. Overall, excluding never-smokers did not materially alter the direction or magnitude of the adjusted estimates, indicating that the main findings are robust.

As a further sensitivity analysis, Firth’s penalised logistic regression was used to assess the robustness of estimates to sparse-data bias, particularly given the small number of never-smokers reporting current e-cigarette use (*n* = 2). Point estimates were consistent with the primary survey-weighted models. Smoking status remained the strongest correlate: the adjusted odds ratio for current smokers compared with never-smokers was 12.19 (95%CI 3.33–44.65, *p* < 0.001) and for ex-smokers was 8.15 (95%CI 2.22–29.97, *p* = 0.002), with substantially narrower confidence intervals than those observed in the main analysis. Older age remained significantly associated with lower odds of e-cigarette use (OR = 0.61, 95%CI 0.43–0.88, *p* = 0.008), and the point estimate for female sex was slightly larger (OR = 1.59, 95%CI 1.03–2.45, *p* = 0.035), though this likely reflects the absence of survey-design adjustment in the Firth model. These results confirm that the key associations were not artefacts of small-cell instability. Interpretively, the markedly tighter confidence intervals on the smoking-status coefficients under Firth’s penalisation, combined with preservation of the direction and rank ordering of point estimates, indicate that the wide intervals observed in the primary survey-weighted model were driven by quasi-complete separation arising from the very small never-smoker user cell rather than by genuine uncertainty in the underlying associations. Taken together with the ever-smoker restricted analysis, the Firth’s estimates support the conclusion that the substantive findings—particularly the strong gradient in e-cigarette use by smoking history, the inverse association with age, and the positive association with deprivation—are not artefacts of sparse cells in the never-smoker stratum.

To assess temporal heterogeneity in the estimated associations across the four pooled survey waves, we tested for interactions between survey year and each of the three principal predictors using design-adjusted Wald tests. No significant interactions were identified: survey year × age (*F* = 0.29, *p* = 0.83), survey year × socioeconomic deprivation (*F* = 1.77, *p* = 0.15), and survey year × smoking status (*F* = 0.56, *p* = 0.69). These results indicate no detectable temporal heterogeneity in the associations of interest and support the inclusion of survey year as a categorical covariate rather than as an effect modifier. We acknowledge, however, that statistical power to detect interaction effects is limited given the modest analytic sample size within each year stratum, and these tests should, therefore, be interpreted as providing no evidence of strong temporal effect modification rather than as positive evidence of temporal stability.

Given that the outcome prevalence (11.9%) was sufficiently high that odds ratios may overstate the magnitude of associations relative to prevalence ratios, we re-estimated the fully adjusted primary model using a modified Poisson regression with survey-design robust variance to obtain prevalence ratios (PRs). All key associations preserved their direction and statistical significance. Older age remained associated with lower prevalence of current e-cigarette use (adjusted prevalence ratio [aPR] = 0.66; 95%CI 0.48–0.90; *p* = 0.009), and socioeconomic deprivation retained a positive gradient (aPR = 1.17; 95%CI 1.01–1.37; *p* = 0.041). Smoking status remained the strongest correlate, with current smokers (aPR = 12.06; 95%CI 2.06–70.71; *p* = 0.006) and ex-smokers (aPR = 9.20; 95%CI 1.53–55.38; *p* = 0.015) showing markedly higher prevalence than never-smokers. As anticipated, PR point estimates were modestly attenuated relative to the corresponding odds ratios; the wide confidence intervals around the smoking-status estimates reflect the very small number of never-smokers reporting current e-cigarette use (*n* = 2), which constrains precision regardless of the estimator used. The substantive conclusions of the analysis were unchanged.

## Discussion

This nationally representative study provides new evidence on the prevalence and determinants of current e-cigarette use among individuals living with chronic obstructive pulmonary disease in Scotland. The weighted prevalence of current use was 11.9%, which is broadly comparable to estimates of e-cigarette uptake reported in UK clinical populations and reflects wider trends in nicotine use among individuals with smoking-related conditions [6, 22]. Consistent with previous research, most current users in this study were either current or former smokers, while use among never-smokers was extremely rare [22–25]. This pattern reinforces that e-cigarettes are predominantly taken up by people with a prior history of combustible tobacco use, often as a harm reduction strategy or as part of attempts to quit smoking [22]. Given the established role of smoking cessation in slowing disease progression and improving outcomes in COPD, understanding these usage patterns is essential for informing targeted cessation support and clinical advice.

Age also emerged as an important determinant of e-cigarette use. The analysis treated age bands as an ordered continuous variable, and older age was associated with lower odds of current e-cigarette use, although polynomial contrast tests indicated some evidence of a non-linear pattern, with the point estimates suggesting the highest odds in the 45–64 age group. This pattern mirrors national trends in e-cigarette uptake, in which younger and middle-aged adults are more likely to use e-cigarettes than those in older age groups [26–28]. These differences may reflect greater familiarity with e-cigarettes, higher technological comfort, and more favourable perceptions of harm reduction tools among younger individuals. In contrast, older adults may be less inclined to adopt newer nicotine devices due to safety concerns, entrenched smoking habits, or limited exposure to e-cigarettes. These findings have clear implications for clinical communication: cessation guidance involving e-cigarettes may need to be tailored by age, ensuring that older adults receive accessible, targeted information that addresses their specific concerns and perceptions.

A key finding of this study is the clear socioeconomic gradient in e-cigarette use among individuals with COPD. Adults living in more deprived areas had significantly higher odds of current e-cigarette use, even after adjusting for demographic and behavioural factors. This is consistent with the wider distribution of COPD in Scotland [1, 29], where disease burden and smoking prevalence are heavily concentrated in socioeconomically disadvantaged communities [30]. E-cigarette use in this context may reflect greater challenges with quitting smoking through conventional methods, variations in perceived accessibility of cessation services, or differing attitudes towards harm reduction products in deprived communities. The UK policy environment, which broadly supports the use of e-cigarettes as a smoking cessation tool, may facilitate transitions away from combustible tobacco, but persistent inequalities suggest that deprivation continues to shape nicotine-use trajectories. These findings highlight the importance of equity-orientated harm reduction interventions that actively address structural barriers to quitting.

The stratified analyses further illuminate differing patterns of behaviour among current and former smokers. Among current smokers, younger age, female sex, and greater socioeconomic deprivation were all associated with current e-cigarette use. This suggests that certain subgroups of smokers with COPD, particularly those facing multiple layers of disadvantage, may be more inclined to use e-cigarettes as a smoking reduction or harm reduction tool. Notably, women comprised a larger proportion of the COPD sample (56.3%) than men, which is consistent with Scottish data showing higher COPD prevalence and incidence among women, particularly in middle-aged groups [31], reflecting the later peak in female smoking prevalence and growing evidence of greater female susceptibility to smoking-related airway disease [32]. The higher odds of e-cigarette use among women who currently smoke may reflect gendered patterns of smoking behaviour, whereby women’s smoking is driven more by behavioural and social cues than by nicotine reinforcement alone [33], combined with evidence that women face greater difficulty achieving sustained cessation [34], suggesting that e-cigarettes may serve as a harm reduction strategy rather than a cessation tool for this group. In contrast, none of the examined sociodemographic or health characteristics were associated with e-cigarette use among former smokers, indicating a more heterogeneous pattern of behaviour once combustible tobacco use has ceased. The smoking intensity analysis adds further nuance: light and moderate smokers had significantly higher odds of current e-cigarette use, whereas heavy smokers did not. This is consistent with emerging evidence that individuals with higher nicotine dependence may find e-cigarettes less satisfying or more difficult to substitute fully for cigarettes [35, 36]. These results suggest that e-cigarettes may play a role for some smokers with COPD, but may be less acceptable or effective for those with heavier patterns of tobacco use.

Taken together, these findings have several implications for policy and practice. First, they underline the importance of integrating tailored harm reduction advice into COPD management pathways, ensuring that healthcare professionals proactively discuss risk and benefits of e-cigarette use. Second, the strong socioeconomic gradient highlights the need for interventions that address structural and financial barriers to smoking cessation in deprived communities, including widening access to cessation services and community-based harm reduction support. Third, the absence of associations between e-cigarette use and broader health or lifestyle factors suggests that e-cigarette use in this population is shaped primarily by smoking history and structural determinants rather than general wellbeing. Finally, while this cross-sectional study cannot determine whether e-cigarette use leads to improved respiratory outcomes or successful long-term cessation, the patterns observed here offer important groundwork for future longitudinal research. Such studies will be critical for understanding whether e-cigarettes can contribute to reducing the substantial health inequalities experienced by people living with COPD.

This study has several strengths. It uses four waves of a nationally representative survey with a robust stratified sampling design, allowing findings to generalise to the adult COPD population in Scotland. The use of survey weights, clustering, and stratification ensures appropriate variance estimation, while the large, pooled sample provides sufficient power for subgroup and stratified analyses. The study also incorporates detailed measures of smoking history, smoking intensity, and sociodemographic factors, allowing a nuanced assessment of determinants of e-cigarette use. Sensitivity analyses, including restriction to ever-smokers, Firth’s penalised logistic regression, and modified Poisson regression to obtain prevalence ratios, confirmed that the key findings were robust to the exclusion of never-smokers, to sparse-data bias, and to the choice of estimator (odds ratio versus prevalence ratio).

However, the study has limitations. Its cross-sectional design prevents any inference about causality or the direction of associations. COPD status is self-reported, which may introduce misclassification, although prior work suggests reasonable validity of self-reported chronic conditions. Such misclassification is most plausibly non-differential with respect to e-cigarette use, since the survey question on e-cigarette use is administered independently of the COPD self-report and there is no obvious mechanism by which an individual’s e-cigarette status would systematically influence their reporting of a COPD diagnosis or vice versa; under non-differential misclassification of a binary exposure, the expected direction of bias is toward the null, such that any true associations would be attenuated rather than spuriously generated. We acknowledge, however, that we did not perform a formal probabilistic quantitative bias analysis, because validation parameters (sensitivity and specificity of self-reported COPD against spirometric or clinically confirmed diagnosis) specific to the Scottish Health Survey are not available, and applying parameters from non-comparable settings could introduce more bias than it resolves. The reported associations should, therefore, be interpreted as conservative estimates of the underlying relationships. Some subgroup estimates, particularly for smaller categories such as light smokers or younger individuals with COPD, have wide confidence intervals reflecting limited precision. Residual confounding is possible, as unmeasured factors, such as nicotine dependence, access to cessation support, or clinician advice, were not available. In addition, data were collected between 2018 and 2022 and may not fully reflect present-day patterns of e-cigarette use, given the rapid evolution of the UK e-cigarette market over this period and the introduction of new product categories. Finally, e-cigarette use was measured as a binary current-use indicator, and, therefore, does not capture frequency, product type, or motivations for use. Despite these limitations, the study provides robust and policy-relevant evidence on patterns of e-cigarette use among individuals with COPD.

## Conclusion

In this nationally representative study of individuals with COPD in Scotland, current e-cigarette use was relatively common and strongly patterned by smoking history, socioeconomic deprivation, and age. E-cigarettes were used predominantly by individuals who continued to smoke or had quit, while use among never-smokers was extremely rare. The socioeconomic and age gradients observed indicate that e-cigarette use is most prevalent among groups already facing substantial tobacco-related and structural disadvantages. These findings highlight the importance of integrating tailored harm reduction advice into COPD care pathways, particularly for smokers who struggle to quit and for those living in more deprived areas. Although further longitudinal research is needed to understand how e-cigarette use influences cessation outcomes and respiratory health over time, the present study provides timely evidence to support equity-focused and clinically informed harm reduction strategies for people living with chronic respiratory disease.

## Data Availability

The Scottish Health Survey data are available through the UK Data Service (https://ukdataservice.ac.uk/find-data/browse/health/), subject to their access conditions.

## Declarations

### Patient and public involvement

Patients and/or the public were not involved in the design, or conduct, or reporting, or dissemination plans of this research.
